# Whole-Genome Identification and Investigation of DNA Methylation Sites in *Nosema ceranae*

**DOI:** 10.3390/biology15040299

**Published:** 2026-02-08

**Authors:** Jianfeng Qiu, He Zang, Kaiyao Zhang, Nian Fan, Yunzhen Yang, Haimei Yue, Dafu Chen, Rui Guo

**Affiliations:** 1 College of Bee Science and Biomedicine, Fujian Agriculture and Forestry University, Fuzhou 350002, China; jfqiu@fafu.edu.cn (J.Q.); zanghe321@163.com (H.Z.); kaiyao1223@fafu.edu.cn (K.Z.); 13595105054@163.com (N.F.); yangyunzhen0120@163.com (Y.Y.); raozhiyou14@163.com (H.Y.); 2 National & Local United Engineering Laboratory of Natural Biotoxin, Fuzhou 350002, China; 3 Apitherapy Research Institute, Fujian Agriculture and Forestry University, Fuzhou 350002, China

**Keywords:** DNA methylation, Oxford Nanopore Technology, *Nosema ceranae*, 5mC

## Abstract

DNA methylation is a key epigenetic modification involved in various biological processes. However, its role in *Nosema ceranae* remains poorly understood. In this study, we employed Oxford Nanopore Technology sequencing to identify whole-genome DNA methylation patterns in *N. ceranae*. A total of 140,711 CpG sites, 170,035 CHG sites, and 1,053,635 CHH sites were detected. Methylation was also observed at varying levels in repetitive genomic regions and different gene regions. Furthermore, three 5mC motifs were identified. These findings provide a foundation for investigation the epigenetic regulatory roles and mechanisms of DNA methylation in *N. ceranae*.

## Simple Summary

*Nosema ceranae* is a fungal parasite that infects honeybees and contributes to colony collapse. However, the role of DNA methylation, an important chemical modification that regulates gene expression, has not been well understood in this organism. In this study, we used advanced sequencing technology to identify DNA methylation patterns across the entire genome of *N. ceranae*. We discovered a significant number of methylation sites in the genome, including different regions associated with genes and repetitive DNA sequences. These findings lay the groundwork for understanding how DNA methylation may affect the behavior and infection process of this parasite. This research is crucial for developing better strategies to control the impact of *N. ceranae* on honeybee populations, which are vital for pollination and global food production.

## 1. Introduction

DNA methylation is a key epigenetic regulatory mechanism present in plants, animals, and microorganisms [1], including *Homo sapiens* [2], *Mus musculus* [3], *Oryza sativa* [4], and *Phytophthora infestans* [5]. DNA methylation is broadly classified into several forms, including N^4^-methylcytosine (4mC), 5-methylcytosine (5mC), and N^6^-Methyladenine (6mA) [6]. In animals and plants, DNA methylation plays essential roles in various processes, such as cellular differentiation, genomic imprinting [7], and embryogenesis [8,9].

Although research on DNA methylation in fungi is still in its early stages, emerging evidence highlights its critical functions in the regulation of gene expression, development, and reproduction. For example, in some fungi, differential DNA methylation has been shown to play a role in regulating hyphal growth and conidiation [10]. *Nosema ceranae*, a single-cell fungal parasite, contributes to honeybee colony collapse [11]. Recent research on *N. ceranae* has encompassed transcriptomics [12], gene function [13], non-coding RNA identification [14,15], and host impact [16]. However, DNA methylation, a critical epigenetic regulation mode in fungi, has not yet been studied in *N. ceranae*.

In this study, we employed third-generation sequencing, Oxford Nanopore Technology (ONT), to conduct whole-genome DNA methylation sequencing on *N. ceranae*, identifying 5mC sites across the genome. Our findings enhance the understanding of fungal DNA methylation and provide a foundation for exploring the epigenetic regulatory roles and mechanisms of DNA methylation in *N. ceranae* proliferation and infection.

## 2. Materials and Methods

### 2.1. Fungi

The *N. ceranae* spores used in this study were previously purified and stored at the Honeybee Protection Laboratory, College of Bee Science and Biomedicine, Fujian Agriculture and Forestry University, Fuzhou, China.

### 2.2. Genomic DNA Extraction and Nanopore Sequencing

Following the procedure outlined by Tourancheau et al. [17], total DNA was extracted from spore samples of the microsporidian infecting the eastern honeybee, utilizing the QIAamp DNA Microbiome Kit (QIAGEN, Hilden, Germany). The extracted DNA was purified using the DNA Clean & Concentrator-5 elution buffer (Zymo Research, Irvine, CA, USA) and ultimately eluted in a solution containing 10 mM Tris-HCl (pH 8.5) and 0.1 mM EDTA. Subsequently, the purified DNA underwent treatment with RNase A. The purity, concentration, and integrity of the DNA were evaluated using a Nanodrop spectrophotometer (Therm Fisher Scientific, Waltham, MA, USA), Qubit 3.0 fluorometer (Therm Fisher Scientific, Waltham, MA, USA), and 0.35% agarose gel electrophoresis. A Nanodrop 2000 spectrophotometer (Therm Fisher Scientific, Waltham, MA, USA) was employed to assess the quality of the input DNA, while a Qubit 3.0 fluorometer was utilized to ascertain DNA concentration. The initial quantity of DNA was set at 1 μg, and the library was assembled using a ligation kit (Oxford Nanopore Technologies, Kidlington, UK), with adapter ligation executed at 24 °C for 30 min. Finally, the samples underwent sequencing on the R9.4.1 MinION platform, employing MinKNOW (version 1.5.12).

### 2.3. Data Quality Control

For quality control of post-sequencing data, processing was conducted in accordance with Zhang et al. [18]: (1) Initially, the multi_to_single_fast5 tool from https://github.com/nanoporetech/ont_fast5_api (accessed on 15 February 2023) was utilized to convert fast5 files into individual files, with each read corresponding to a separate fast5 file. (2) Guppy 5.0.16 software was employed for base calling with the dna_r9.4.1_450bps_hac_modbases_5mC_6mA.cfg model, converting fast5 format data to fastq format. (3) The fastq data was further filtered to remove adapters, short fragments (length < 500 bp), and low-quality reads, yielding high-quality clean reads for subsequent analysis. Subsequently, clean reads were aligned to the reference genome of the microsporidian (assembly ASM98816v1, *Nosema ceranae* BRL01) employing the Split-Reads program of Minimap2 software (2.25). Through analysis of clean read locations on the reference genome, sequencing depth and alignment efficiency were assessed.

### 2.4. DNA Methylation Site Detection

To ascertain DNA methylation status, Nanopolish software (0.13.2) [19] was employed to detect CpG methylation through a hidden Markov model. Specifically, the nucleotide string on the reference genome to be analyzed for methylation status is designated as SR (this sequence must contain at least one CpG site and its five adjacent bases). The methylation status of the SR region was determined by aligning base-called reads to the reference genome and assessing the probability of coverage within the SR region. To define a CpG site as methylated, we applied two strict thresholds: Minimum read coverage ≥ 10× and Log-likelihood ratio (LLR) cutoff ≥ 2.0. Only CpG sites meeting both criteria were retained for analysis. Tombo software (v1.5.1) [20] was utilized to identify CHH (H = A/T/C) and CHG sites. The Tombo software (v1.5.1) first performs re-squiggle on the sequencing data and then uses the Alternative Model to detect CHH and CHG sites. Minimum read coverage ≥ 10×; LLR cutoff ≥ 3.0 (a higher LLR cutoff was used to minimize false positives for low-frequency non-CpG methylation events). Additionally, we filtered out CHG/CHH sites with a methylation frequency below 10% to exclude stochastic methylation signals.

### 2.5. Analysis of DNA Methylation Levels

Chromosomes were segmented into 100 Kb windows to calculate average methylation levels, displayed genome-wide. RepeatMasker software (4.1.4) [21] was used to predict repeat regions, which, along with flanking regions (2 Kb upstream and downstream), were divided into 50 bins for average methylation level calculations. Methylation distribution was analyzed around the transcription start site (TSS) and transcription termination site (TTS), considering the influence on gene transcription. The average methylation levels were plotted, with regions divided into 50 bins for detailed analysis.

### 2.6. Analysis of Sequence Features

According to Zhang et al. [22], the 1000 CpG, CHG, and CHH sites with the highest methylation levels were selected for sequence feature analysis using weblogo software (3.7.12).

## 3. Results

### 3.1. High-Quality Sequencing Data and Genome-Wide DNA Methylation Profiling of N. ceranae

**Figure 1:**
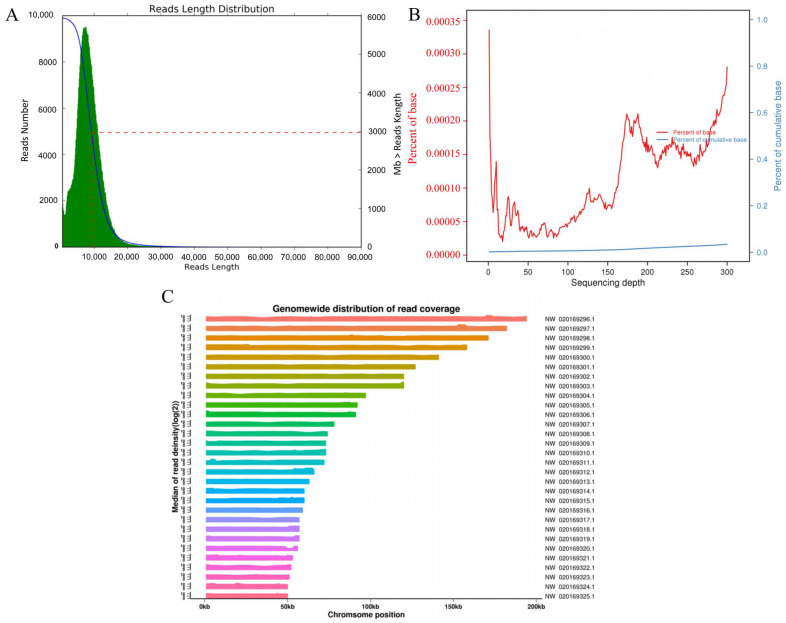
Data quality and genome-wide methylation levels. (**A**) Length distribution of clean reads. The horizontal axis represents the read length (bp); the left vertical axis indicates the number of reads (green); the right vertical axis represents the total base count (Mb) contained in reads longer than the corresponding length (blue); the red dashed line indicates the N50 length of the reads. (**B**) Sequencing saturation curve. The horizontal axis represents sequencing depth; the left vertical axis indicates the percentage of bases corresponding to that depth (red curve); the right vertical axis indicates the cumulative percentage of bases at or below that depth (blue curve). (**C**) Chromosome distribution of clean reads. The horizontal axis represents chromosomal position, and the vertical axis indicates the coverage depth (log_2_) at the corresponding chromosomal locus.

In this study, we obtained 726,558 clean reads, with an N50 value of 9233 bp and an N90 value of 5584 bp. The average read quality was 8.96, and the average length of clean reads was 8175 bp (Figure 1A). The clean reads aligned to the reference genome (assembly ASM98816v1, *Nosema ceranae* BRL01) with an alignment rate of 95.75%, indicating high data quality suitable for subsequent analysis. Furthermore, genome coverage increased concomitantly with sequencing depth, confirming that the achieved depth was sufficient for the detection of numerous methylation sites (Figure 1B). Clean reads were distributed across all *N. ceranae* chromosomes, exhibiting the highest read distribution on contig NW_020169296.1 and the lowest on NW_020169325.1, which indicated good sequencing randomness (Figure 1C). Following quality control, we identified a total of 140,711 CpG, 170,035 CHG, and 1,053,635 CHH methylation sites.

### 3.2. Profiling of 5mC Methylation in Repetitive Regions, Gene Elements, and Sequence Motifs

**Figure 2:**
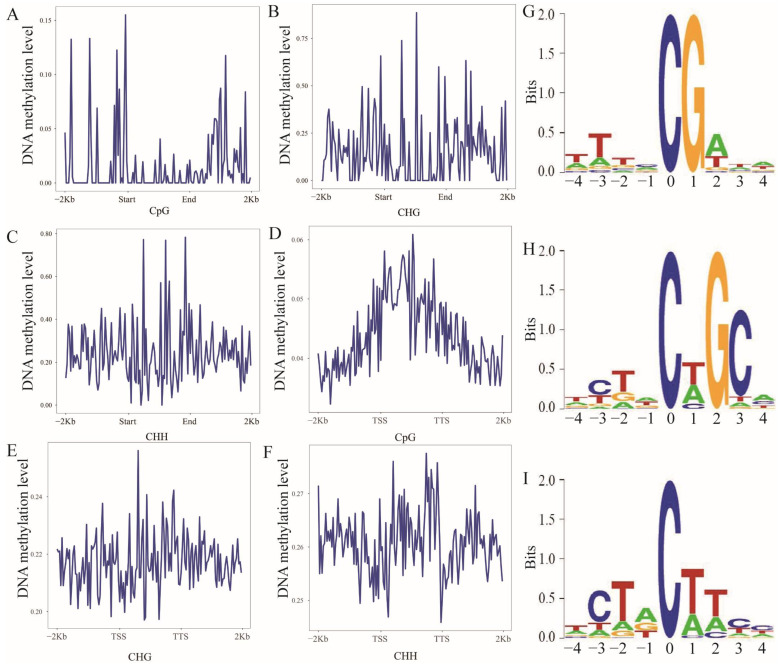
Methylation levels of CpG (**A**), CHG (**B**), and CHH (**C**) in repetitive regions. Methylation levels of CpG (**D**), CHG (**E**), and CHH (**F**) in different gene regions. Distribution of the 9 bp sequence around the 5mC site. Select the 1000 CpG (**G**), CHG (**H**), and CHH (**I**) sites with the highest methylation levels respectively, and use the weblogo software (3.7.12) to statistically analyze the sequence characteristics of the 9 bp region surrounding the C site. The horizontal axis represents the number of bases at the methylation site, the total height at each position indicates the sequence conservation of the base at that location, and the height of the base signal reflects the relative frequency of the base at that position.

The methylation levels displayed a broad dynamic range: CpG averaged 0.015 (range: 0.002–0.155), CHG averaged 0.15 (range: 0.0258–0.8853), CHH averaged 0.24 (range: 0.0099–0.78295 per bin (Figure 2A–C). Strikingly, analysis of gene regions showed that the gene body exhibited the highest methylation levels in all three sequence contexts (CpG, CHG, and CHH) (Figure 2D–F). For CpG, CHG, and CHH, the average methylation levels per bin were 0.22, 0.26 and 0.044, respectively (Figure 2D–F). We further analyzed the 9-bp sequence features surrounding 5mC sites and found that the base adjacent to the methylated cytosine at CHG and CHH sites was T[A/C]G and T[A/C]T[A/C], respectively (Figure 2G–I).

## 4. Discussion

Epigenetics is crucial for regulating gene expression, with DNA methylation being a primary modification mode [23]. Whole-genome DNA methylation has been studied in various species, including *H. sapiens* [24] and *O. sativa* [25]. In this study, we explored DNA methylation in *N. ceranae*, identifying 140,711 CpG, 170,035 CHG, and 1,053,635 CHH sites.

DNA methylation in CpG islands, CHH, and CHG often inhibits genomic DNA by producing methylated repeat sequences, protecting the genome [26]. However, Nosema ceranae, as a fungal parasite, lacks homologs of key methylation-related enzymes (e.g., DRM2 and CMT3) present in other species. In the early stage, we cloned Methyltransferase-like protein 5 (Mettl5) and N6-adenine-specific methyltransferase (N6AMT) which are related to methylation in *N. ceranae* [27,28]. Nevertheless, it is still uncertain whether *N. ceranae* possesses a methylation mechanism.

We observed higher CHH and lower CpG methylation levels in repetitive regions, consistent with chromosomal patterns. This indicates cytosine methylation mainly occurs in CHG and CHH contexts in repeats, confirming enrichment in DNA repeat sequences [29].

The TSS region is crucial for gene expression regulation [30]. Methylation in this region is associated with transcriptional silencing [31]. While our study identified 5mC methylation around the TSS and gene body regions, suggesting potential roles in gene regulation, we acknowledge that these patterns alone do not provide direct evidence of their functional significance. In other words, no functional correlation was established between the observed methylation and gene expression, which is a limitation of this study. 5mC is a common modification in eukaryotic genomes, especially in CpG dinucleotides [32]. We identified 5mCpG, 5mCHG, and 5mCHH motifs in *N. ceranae*, indicating conservation across species.

## 5. Conclusions

In conclusion, using ONT sequencing, we identified a wide range of methylation sites in *N. ceranae*, providing a foundation for further exploration of the epigenetic roles and mechanisms of DNA methylation in this organism. In future research we will explore the role of DNA methylation in *N. ceranae* virulence and its interactions with honeybee hosts.

## Data Availability

The raw sequencing data have been uploaded and are available at: https://www.ncbi.nlm.nih.gov/sra/?term=SRR37000696 (accessed on 18 December 2025).
